# Application of controlled release urea improved grain yield and nitrogen use efficiency: A meta-analysis

**DOI:** 10.1371/journal.pone.0241481

**Published:** 2020-10-29

**Authors:** Shuhao Zhu, Liyuan Liu, Yan Xu, Yanying Yang, Rongguang Shi

**Affiliations:** Agro-Environmental Protection Institute Ministry of Agriculture and Rural Affairs, Tianjin, China; University of Helsinki, FINLAND

## Abstract

The application of controlled release urea (CRU) has been proposed as a crucial method to reduce the adverse environmental effects induced by conventional urea (CU). Yet, a systematic and quantitative analysis on how CRU affects staple crop production including wheat (*Triticum aestivum L*.), maize (*Zea mays L*.), and rice (*Oryza sativa L*.) is lacking. Here, a meta-analysis was conducted to determine how CRU influences soil chemical properties, total nitrogen (TN) uptake, grain yield, and nitrogen use efficiency (NUE) of staple crop in China. The results indicated that CRU application significantly increased soil organic carbon (SOC), TN, and available nitrogen (AN) by 5.93%, 3.89% and 13.98% respectively overall, while soil pH showed no significant changes. Compared to the application of CU, applying CRU significantly increased grain yield by 7.23%, which was mainly owing to the higher TN uptake (9.13%) across all the studies. In addition, the application of CRU significantly increased NUE, nitrogen agronomy efficiency (NAE), utilization rate of nitrogen fertilizer (NUR), and nitrogen physiological efficiency (NPE) by an average of 23.4%, 34.65%, 25.83% and 15.8% respectively which could be attributed to the slow nitrogen (N) release characteristics of CRU. The positive effect of CRU on grain yield and NUE of staple crop was greatest when the content of SOC and TN were extremely low, indicating that it was most effective to improve grain production of infertile soil by applying CRU. The finding of this study indicated that the application of CRU should be promoted for grain production, especially for infertile soil.

## Introduction

Feeding the ever-growing population without further damaging the environment is the grand challenge in the 21^th^ century [1]. As the world’s biggest nitrogen (N) fertilizer producer and consumer, China plays an indispensable role in world food production [2]. The amount of fertilizer N applied on staple crops, namely wheat (*Triticum aestivum L*.), maize (*Zea mays L*.), and rice (*Oryza sativa L*.) were nearly 16.2 Tg which made up more than 60% of the total fertilizer N for food production in the past years in China [3]. However, about 20–50% of the fertilizer N was lost as greenhouse gas and other reactive N species, which has triggered serious environmental damage including widespread soil acidification [4], serious greenhouse effect [5] and devastating water pollution [6]. With the increasing public attention to environmental issues, Ministry of Agriculture and Rural Affairs of China formulated a policy of zero growth in chemical N fertilizer application by 2020 [7]. Consequently, reducing chemical N fertilizer application while improving grain yield simultaneously is the primary object for agriculture production in China.

Numerous researches have well indicated that grain yield was not only influenced by the amount of fertilizer N application but also N fertilizer management practices [8,9]. Improved N fertilizer management practices which could enhance crop yield and decrease environmental footprint are urgently demanded in China. Studies have demonstrated that crop growth required continuous N supply but the amount of N absorbed by crops under different growth stages was not exactly the same [10]. In terms of rice, the maximum N demand was appeared at the grain filling period, while there was little N absorption at the seeding stage [11]. However, conventional urea (CU) increased soil available N content rapidly after the application in two weeks, which led to N deficiency at late growth period [12]. Although increasing splitting frequency of fertilizer application could increase N use efficiency (NUE) and crop productivity, it was much more time-consuming and laborious than one-time fertilization [13].

Controlled release urea (CRU) could control the release rate of N by the functional materials added to urea which has been widely used in China in recent years [14,15]. For example, resin-coated urea and sulfur-coated urea control the N release rate by coating on the surface of urea granules with resin and sulfur respectively [16,17]. Great achievements have been obtained about the application of CRU in crop production. For instance, the study of Grant et al (2012) demonstrated that CRU could reduce N loss and realize one-time fertilization, so as to increase NUE and wheat yield [18]. However, there was also study indicated that the application of CRU at 240 kg N ha^–1^ decreased maize yield compared to the treatment with CU at 240 kg N ha^–1^ [19]. Therefore, clarifying the factors controlling the discrepancy in crop production and NUE response to the application of CRU is vital to encourage the development and promotion of the utilization of CRU.

Meta-analysis is a powerful statistical technique for integrating the results of independent experiments to quantitatively assess the direction and magnitude of a treatment effect and detect the underlying factors on global and regional scales [20,21]. The present study is a meta-analysis of field experiments about the response of grain yield and NUE of staple crop to the application of CRU in China which aims to reveal: (i) how soil chemical properties, including pH, soil organic carbon (SOC), total nitrogen (TN), and available nitrogen (AN) respond to CRU application, (ii) how TN uptake and grain yield are affected by applying CRU, (iii) how NUE, nitrogen agronomy efficiency (NAE), utilization rate of nitrogen fertilizer (NUR), and nitrogen physiological efficiency (NPE) respond to CRU application, and (iv) how the effects varies of grain yield and NUE regarding SOC and TN content.

## Material and methods

### Data collection

A collection of peer-reviewed articles published before April 2020, which concentrated on the responses of soil chemical properties (pH, SOC, TN, and AN), grain yield (wheat, maize, and rice), TN uptake (Dry weight in aboveground part of plant × N content in aboveground part of plant), NUE ((N uptake of aboveground plants in N application area—N uptake of aboveground plants in non-N application area) / N application rate), NAE ((Yield in N application area—Yield in non-N application area) / N application rate), NUR (TN uptake in N application/TN application), and NPE ((Yield in N application area—Yield in non-N application area) / (N uptake of aboveground plants in N application area—N uptake of aboveground plants in non-N application area)) to CRU application in China. Data published in English were collected from the Web of Science (https://www.webofknowledge.com/), Science Direct (https://www.sciencedirect.com/), and Springer Link (https://www.springerlink.com/), and data published in Chinese were collected from the China Knowledge Resource Integrated Database (https://www.cnki.net/). The search terms applied for the present study were “Controlled release urea”, “Grain yield”, “Total nitrogen uptake”, “Nitrogen use efficiency”, and “Soil chemical properties”.

To prevent data distortion during the literature collection, the publications chosen for data analysis had to meet the following criteria: (i) the studies had to be conducted in the field instead of in a pot or greenhouse; (ii) the experiment sites had to be located in China; (iii) the studies had to be conducted with side-by-side comparisons of control (without CRU application) and treatment (with CRU application) groups; (iv) the means, standard deviations (*SD*), and sample sizes of the observations had to be reported or had the possibility of being calculated. If standard errors (*SE*s) were provided in the paper, *SDs* were calculated using

$$SD=SE\times \sqrt{n}$$ (1)

Where *n* was the replicate number. If soil organic matter (*SOM*) was reported, the SOC was calculated by the equation:

SOC=SOM×0.58 (2)

We assumed that the *SD* was one-tenth of the mean in cases where there was no *SE* or *SD* reported [22]; If the variables concerning soil chemical properties included more than one soil layer, only the uppermost layer was used in the present research.

**Fig 1:**
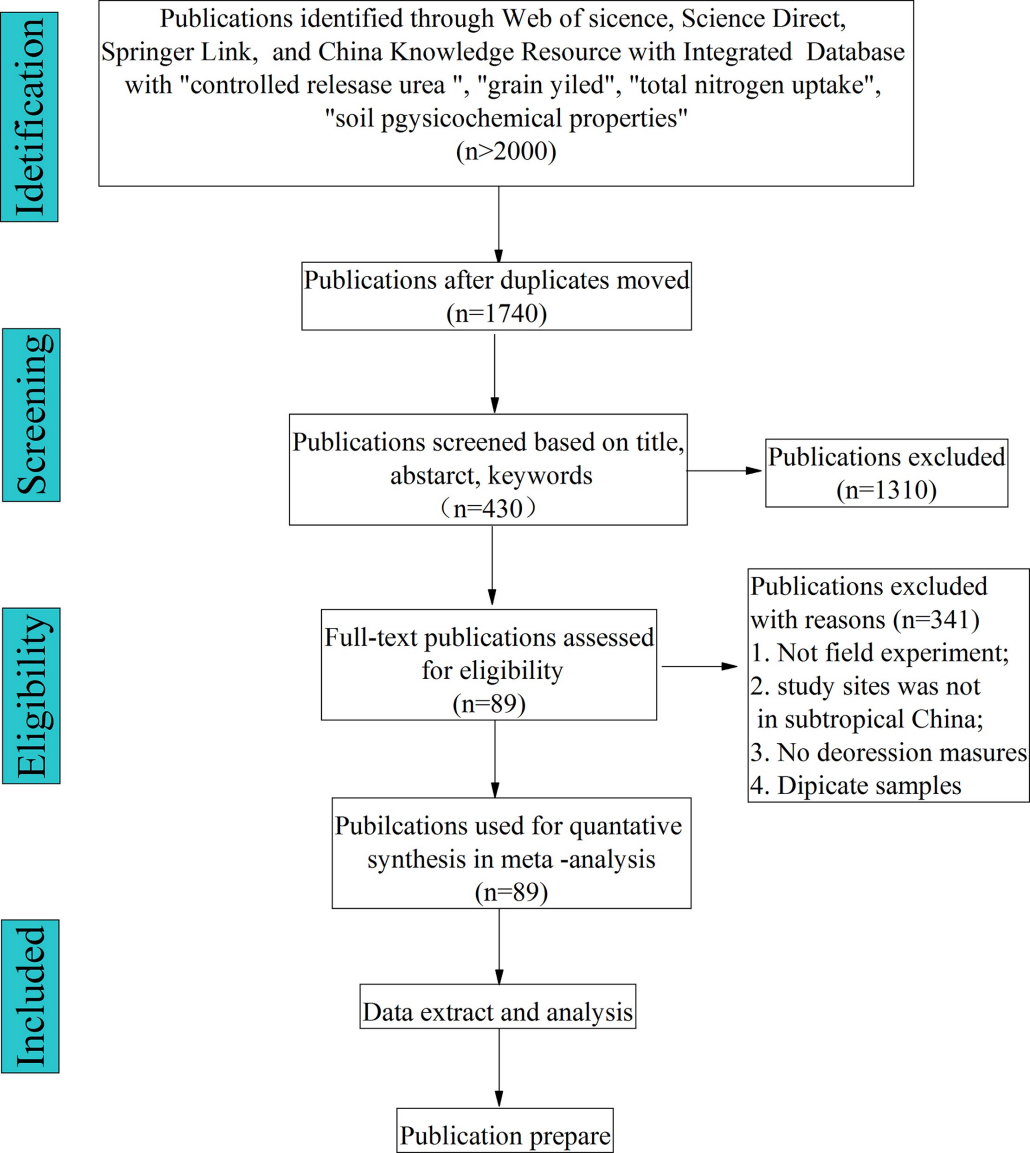
Flow chart for selection of publications.

**Fig 2:**
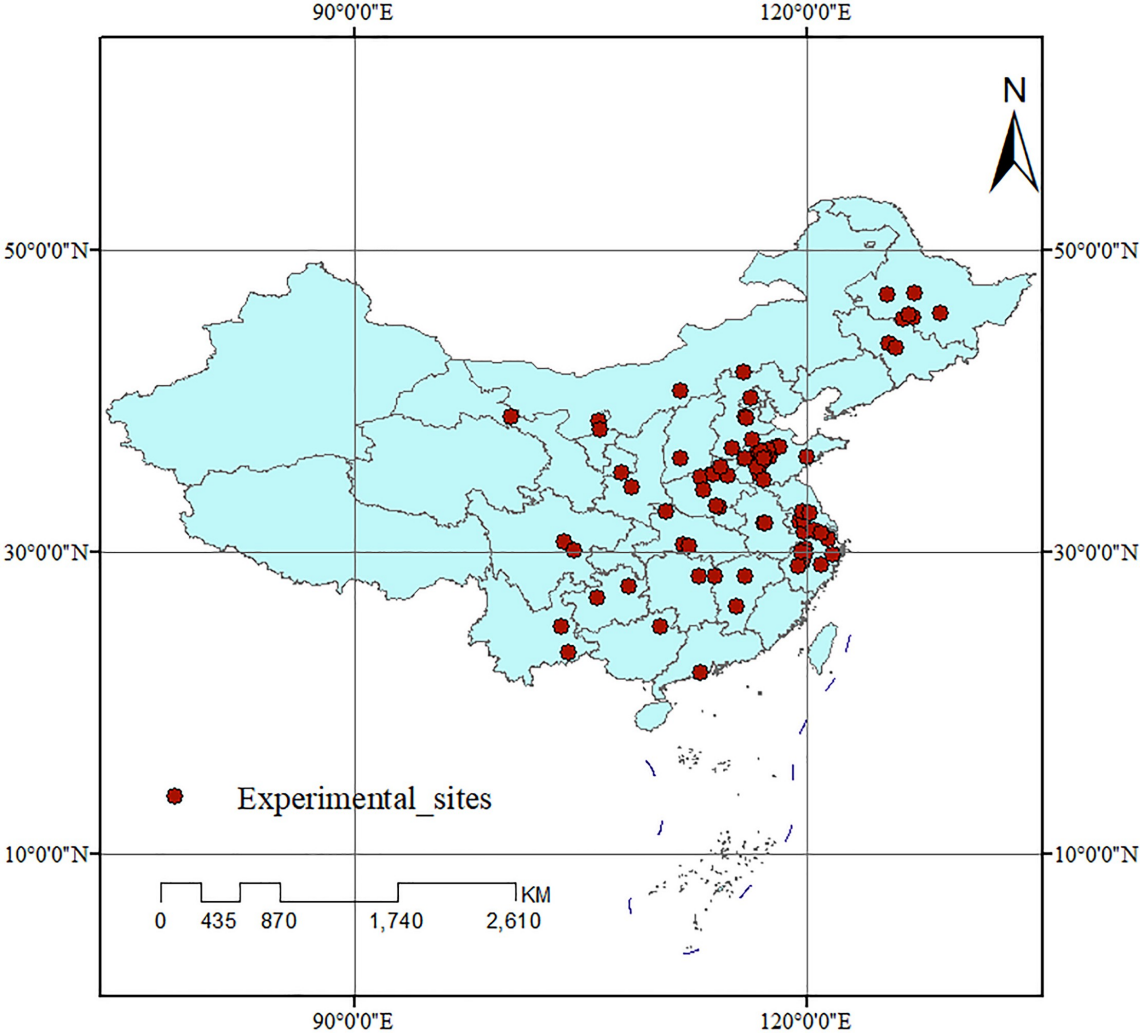
Locations of the field experiment sites in China that was included in this meta-analysis.

After the filtering procedure, as shown in Fig 1, 89 peer-reviewed papers from 99 sites in which the soil texture almost were loam in China were selected. The geographic distribution of experimental sites and detailed information were shown in Fig 2 and S1 Table (Appendix material), respectively. For each collected paper, we recorded the soil chemical properties, grain yield, TN uptake, and NUE, NAE, NUR, and NPE. For data displayed graphically, GetData Graph Digitizer version 2.24 was applied to digitize the data.

To further explore the variation in grain yield induced by CRU application, the experimental data were partitioned into two subcategories according to the content of SOC and TN: (i) SOC—extremely low (< 10 g·kg^-1^), low (10–15 g·kg^-1^), moderate (15–20 g·kg^-1^), and fertile (> 20 g·kg^-1^); and (ii) TN—extremely low (< 1 g·kg^-1^), low (1–1.5 g·kg^-1^), moderate (1.5–2 g·kg^-1^), and fertile (> 2 g·kg^-1^).

### Data analysis

The response ratio (*RR*) is an index that is used to evaluate the effect of the experiment on each variable in the meta-analysis [23]. For a given variable, *RR* is determined as the ratio of the mean value of the treatment group (*M*_*t*_) to that under the control group (*M*_*c*_). The calculation formula of *RR* is as follows:

$$RR=\frac{M_{t}}{M_{C}}$$ (3)

where *M*_*t*_ and *M*_*c*_ represent the mean values of the treatment groups and control groups, respectively.

The ln*RR* is the natural logarithm of *RR*. It indicates a positive effect of CRU fertilization on a variable if the value of ln*RR* is above 0; however, a negative effect of CRUertilization is exhibited when ln*RR* is below 0. The ln*RR* was estimated using

$$\ln RR=\ln\frac{M_{t}}{M_{C}}=\ln M_{t}-\ln M_{C}$$ (4)

The variance (V) was calculated by:

$$V=\frac{SD_{t}^{2}}{n_{t}M_{t}^{2}}+\frac{SD_{c}^{2}}{n_{c}M_{c}^{2}}$$ (5)

Where n_t_ and n_c_ represent the sample sizes of the treatment and control groups, respectively, and *SD*_*t*_ and *SD*_*c*_ represent the *SD* values of the organic fertilization treatments and control groups, respectively.

In addition, the weighted factor (*W*_ij_), weighted response ratio (*RR*_++_), standard errors of *RR*_++_ (*S*(*RR*_++_)), and 95% confidence intervals (95% *CIs*) were calculated by

$$W_{ij}=\frac{1}{V}$$ (6)

$$RR_{++}=\frac{\sum_{i=1}^{m}\sum_{j=1}^{ki}W_{ij}\ln RR_{ij}}{\sum_{i=1}^{m}\sum_{j=1}^{ki}W_{ij}}$$ (7)

In this study, *RR*_++_ was calculated as *RR*_*++*_×100%:

$$S\left(RR_{++}\right)=\frac{1}{\sqrt{\sum_{i=1}^{m}\sum_{j=1}^{ki}W_{ij}}}$$ (8)

$$95\%CI=RR_{++}\pm 1.96S\left(RR_{++}\right)$$ (9)

The responses of the variable to CRU application significantly (*p* < 0.05) differed from the control if the 95%*CI* value of *RR*_*++*_ for a given variable did not cross zero [24].

## Results

### Soil chemical properties

**Fig 3:**
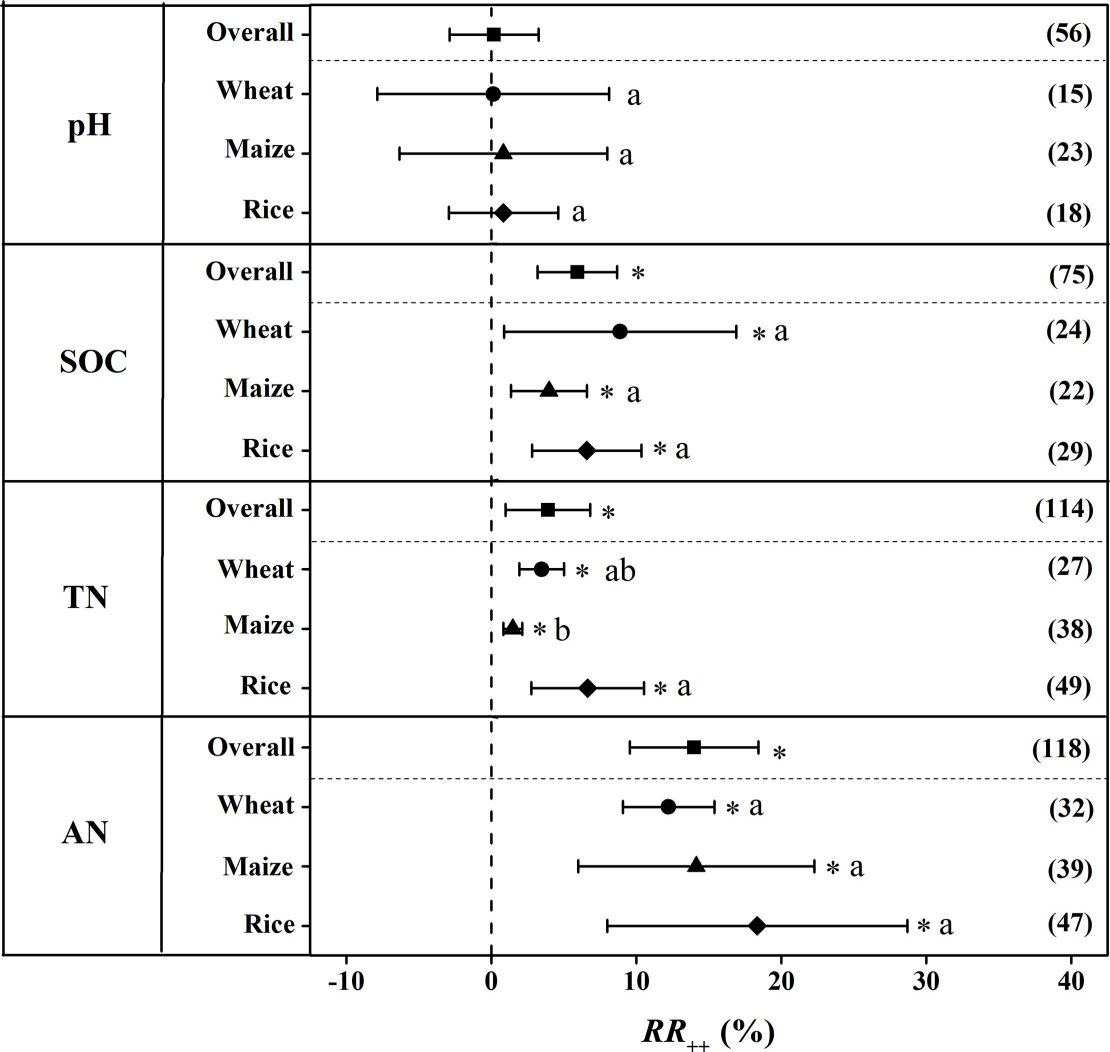
The weighted response ratio (*RR*_*++*_) for the response of soil chemical properties to CRU application for different staple crops. **Error bars represented 95% confidence intervals (CI).** The numbers were the sample size for each variable. The star (*) indicates significance when the 95% CI that do not go across the zero line. CRU: controlled release urea, SOC: soil organic carbon, TN: total nitrogen, AN, available nitrogen.

The overall responses of selected soil chemical properties to CRU application was presented in Fig 3. Average across all studies, the effect of CRU application on soil pH was not significant compared to the application of CU. Responses of soil pH to a certain specific crop including wheat, maize, and rice were all not significant to the application of CRU. On average, SOC was significantly increased by 5.93% to CRU application. The improvement of CRU application on SOC in wheat, maize and rice was 8.87%, 3.97% and 6.58% respectively with no significant difference among each other. Compared to the treatment with CU application, CRU application significantly increased soil TN content by 3.89% overall. The increase in soil TN content with CRU was largest in rice (6.65% 95%*CI*: 2.77%-10.53%), followed by wheat (3.47%, 95%*CI*: 1.94%-5.01%), and maize (1.47%, 95%*CI*: 0.82%-2.12%). Averaged across all the studies, the content of AN under the treatment with CRU application significantly increase by 13.98% (95%*CI*: 9.55%-18.41%). The increase of soil AN content with CRU in rice, wheat and maize was 18.83%, 12.23% and 14.13% respectively with no significant difference among each other (Fig 3).

### Grain yield and total N uptake

**Fig 4:**
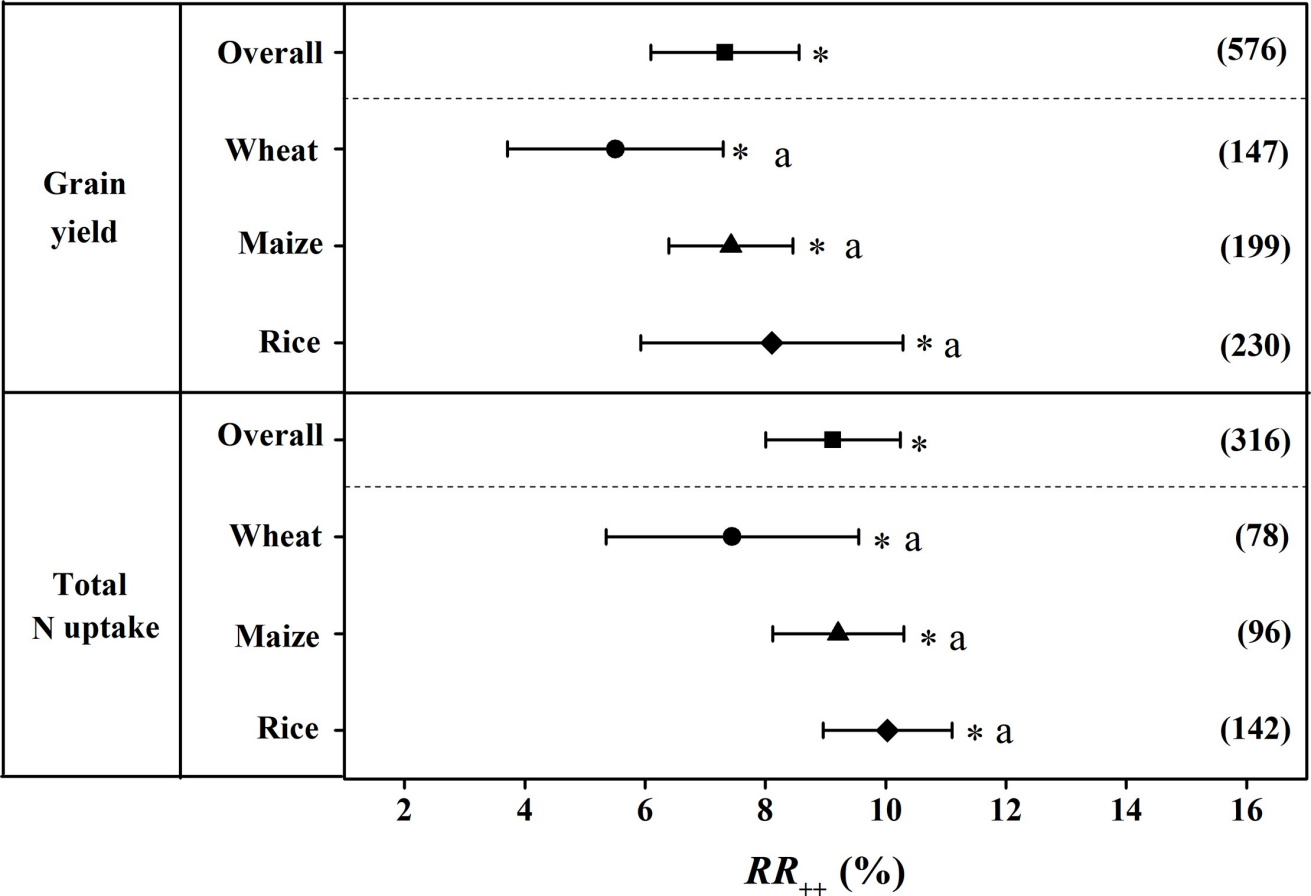
The weighted response ratio (*RR*_*++*_) for the response of grain yield and TN uptake to CRU application for different staple crops. Error bars represented 95% confidence intervals (CI). The numbers were the sample size for each variable. The star (*) indicates significance when the 95% CI that do not go across the zero line. TN: total nitrogen, CRU: controlled release urea.

Across all the studies, grain yield was significantly increased by 7.32% for CRU application compared to CU application (Fig 4). There was no significant difference about the increase in grain yield to CRU application in rice, wheat and maize. Specifically, the grain yield was significantly increased by 8.11%, 5.5% and 7.42% for rice, wheat, and maize respectively. Overall, TN uptake on average significantly increased by 9.12% for CRU application (Fig 4). The effect of CRU application on TN uptake was 10.03%, 7.45%, and 9.21% respectively with no significant difference among each other.

### NUE, NAE, NUR, and NPE

**Fig 5:**
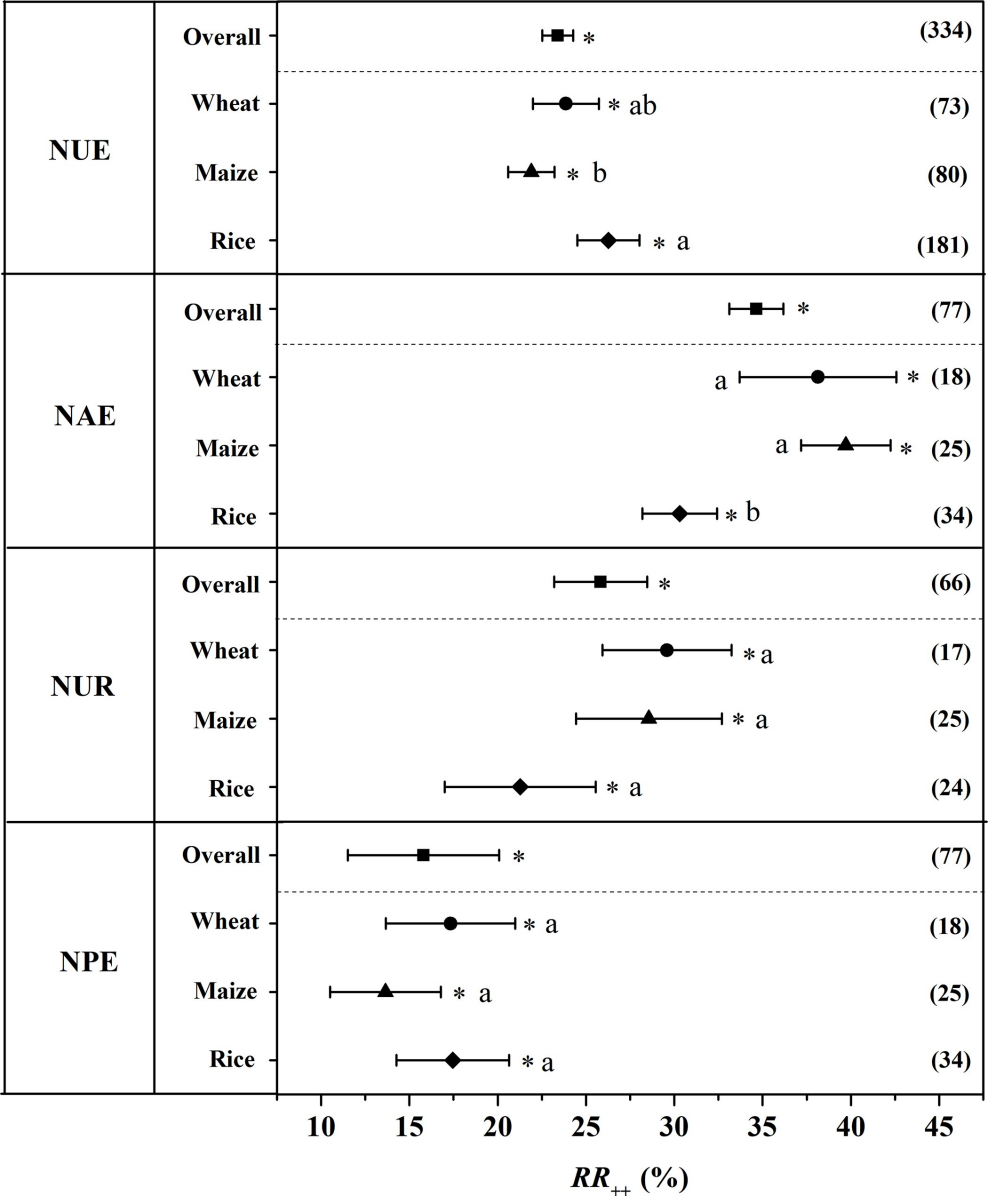
The weighted response ratio (*RR*_*++*_) for the response of NUE, NAE, NUR and NPE to CRU application for different staple crops. Error bars represented 95% confidence intervals (CI). The numbers were the sample size for each variable. The star (*) indicates significance when the 95% CI that do not go across the zero line. NUE: Nitrogen use efficiency, NAE: Nitrogen agronomy efficiency, NUR: Utilization rate of nitrogen fertilizer, NPE: Nitrogen physiological efficiency, CRU: Controlled release urea.

Overall, the NUE was significantly enhanced by 23.4% for the application of CRU compared to the application of CU (Fig 5). The increased of NUE was greatest in rice (26.27%), followed by wheat (23.87%) and maize (21.91%). Moreover, the increase of NUE in rice was significantly higher than that in maize.

NAE also showed a positive response to the application of CRU, with an increase of 34.65% across all the studies (Fig 5). The greatest improvement of NAE to CRU application was observed in maize, with an increase of 39.71% (Fig 5). A stronger increase of NAE was observed in wheat (38.14%) than that in rice (30.31%) for applying CRU.

Compared to the treatment with CU application, the NUR was significantly increased by 25.83% with CRU application on average (Fig 5). The application of CRU resulted in a biggest increase of NUR in wheat with 29.59%. Following wheat, the application of CRU increased NUR by 28.56% and 21.28% in maize and rice respectively.

Overall, NPE showed a significant increase to the application of CRU by 15.8%. The positive effect of CRU application on NPE was 17.46%, 17.33%, and 13.65% in rice, wheat and maize respectively to the application of CRU as shown in the Fig 5.

### Grain yield and NUE at different SOC and TN content

**Fig 6:**
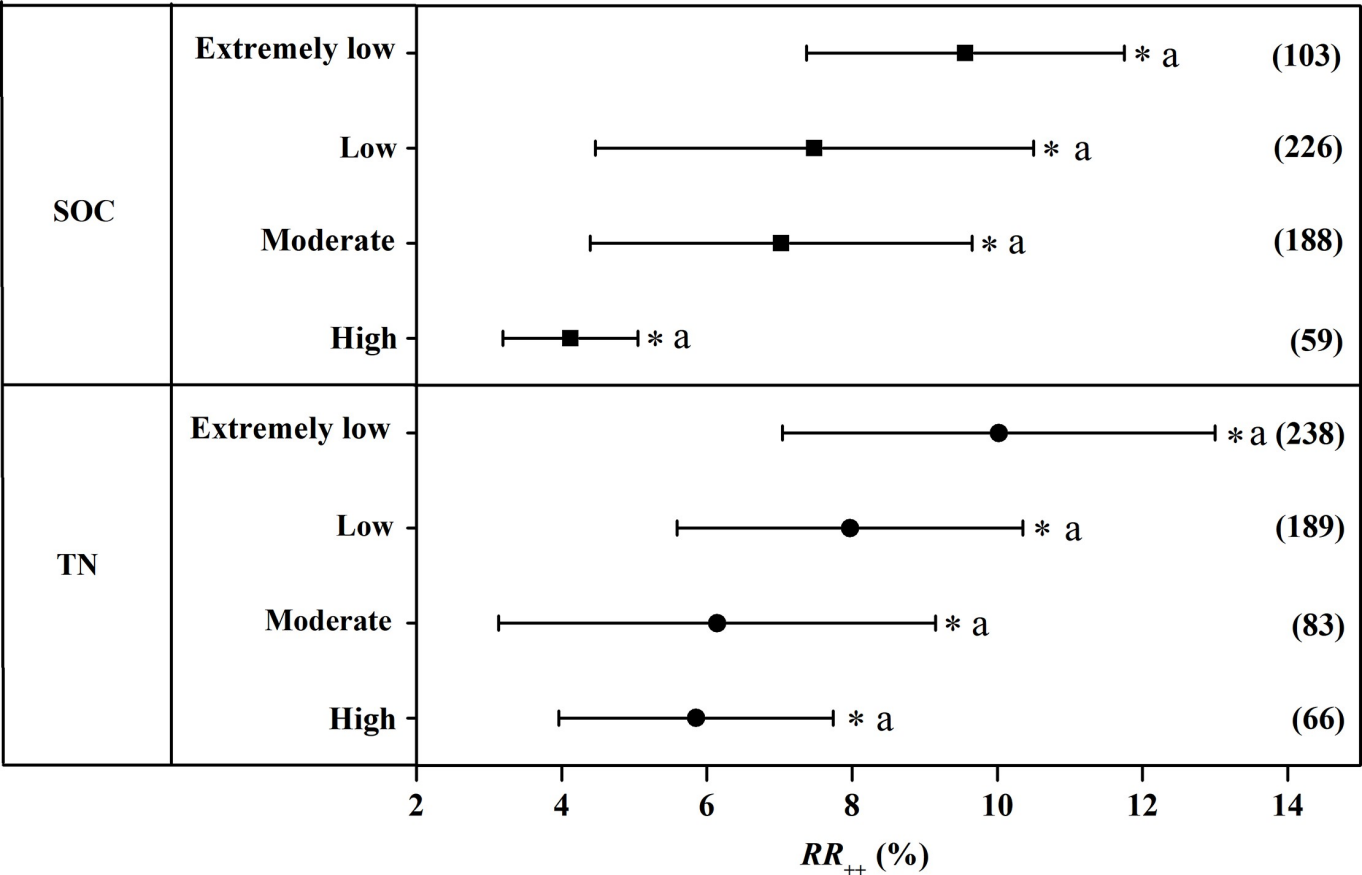
The weighted response ratio (*RR*_*++*_) for the response to CRU application of grain yield at different SOC and TN content. Error bars represented 95% confidence intervals. The numbers were the sample size for each variable. CRU: Controlled release urea, SOC: Soil organic carbon, TN: Total nitrogen.

The variation in grain yield and NUE induced by CRU application at different SOC and TN content was shown in Fig 6. The positive effect of CRU application on grain yield showed a downtrend with the increase of SOC. Specifically, the increase of rice yield with CRU application was greatest when SOC was extremely low with an increase of 9.56%. The magnitude of the increase in grain yield to CRU application when SOC was low and moderate was 7.48% and 7.02% respectively. The least increase of grain yield with CRU fertilization was observed when SOC was high with an increase of 4.12%.The response ratio of grain yield to CRU application tended to decline with the increase in soil TN content (Fig 6). CRU application resulted in a significant increase of 10.02%, 7.97%, 6.14%, and 5.85% in grain yield in the soil with extremely low, low, moderate and high TN content respectively with no significant difference among each subclass.

**Fig 7:**
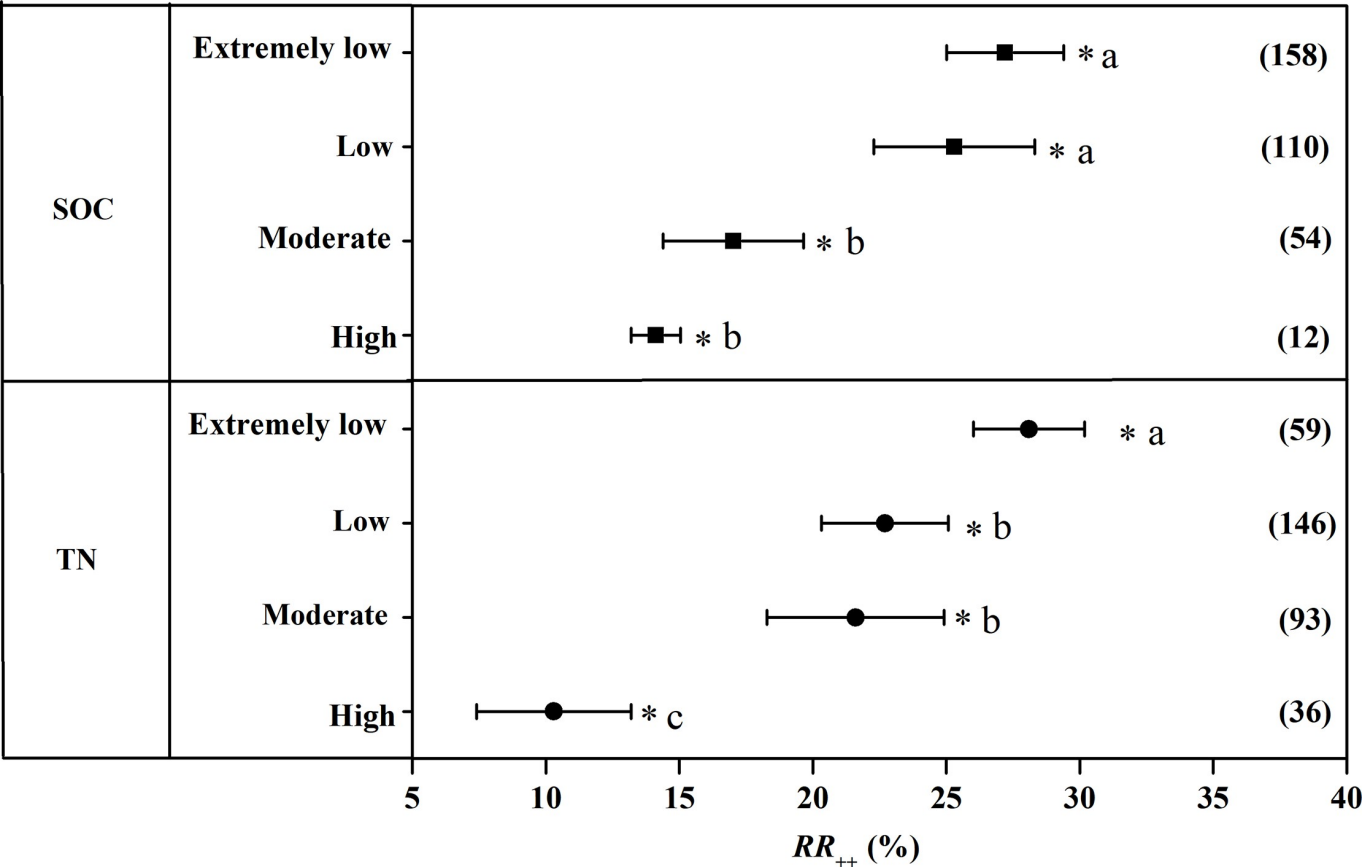
The weighted response ratio (*RR*_*++*_) for the response to CRU application of NUE at different SOC and TN content. Error bars represented 95% confidence intervals. The numbers were the sample size for each variable. NUE: Nitrogen use efficiency, CRU: Controlled release urea, SOC: Soil organic carbon, TN: Total nitrogen.

Similar with grain yield, the increase of NUE to CRU application also showed a downtrend with the increase of SOC (Fig 7). The increase of NUE to the application of CRU was highest when SOC was extremely low with an increase of 27.2%. And applying CRU increased NUE by 25.3% when SOC was low. The increase magnitude of NUE with CRU application was 17.02% and 14.12% when SOC was moderate and fertile respectively. Regarding soil TN content, the increase of NUE with CRU application was greatest when soil TN content was extremely low (28.1%) which was significantly higher than that in the other three subclasses. The magnitude of the increase in NUE to CRU application when soil TN content was low and moderate was 22.7% and 21.6% respectively. The increase of NUE with CRU fertilization was least when soil TN content was high with an increase of 10.3%.

## Discussion

Soil is of great importance to crop production and its quality could be best assessed by soil chemical properties. In the present study, the application of CRU had no significant influence on soil pH, which has been demonstrated in previous study [25]. Applying CRU significantly increased SOC compared to CU as shown in the Fig 3, which might be induced bythe increased input of SOC to soil via crop residues including fallen leaves and roots with the improvement of crops growth by CRU application [26]. Moreover, soil TN and AN also showed positive response to the application of CRU in the present study (Fig 3). Two reasons might be given for the increase of soil TN and AN content to CRU application in staple crops production. Firstly, similar with SOC, CRU facilitate in the growth of crops especially in the advanced stages which lead to more grain yield and biomass, and also increased residues and consequently increase soil TN and AN [27]. Moreover, it has been demonstrated that CRU significantly decreased N runoff and leaching, nitrous oxide emission and ammonia volatilization [28].

The results of the present study demonstrated that the positive effects of CRU on grain yields and NUE compared with CU which was consistent with the results of previous studies [29–31]. The main reason why CRU increase grain yield and NUE may involve its N release characteristics [32]. In general, CRU releases N over several months which could satisfy the N requirement by crops over the whole growing period [33]. In detail, CRU can minimize early season N availability when crops uptake is low, and increase N availability during the advanced stage when the demand for N of crops is high [34]. In addition, numerous studies have indicated that the application of CRU could decrease reactive N losses. For example, Liu et al. (2016) have reported that N runoff and leaching, ammonia emission was significantly decreased by 20–50% and 17–32% respectively under the treatment to CRU compared with CU due to the slow release of N from CRU [35]. The decreases of reactive N loss rate to the environment subsequently improve NUE. Consequently, it is effective to increases grain yield and increases NUE by applying CRU. Moreover, the effect of CRU on grain yield and NUE was stronger in rice than wheat and maize as shown in Figs 4 and 5 respectively in our study.

The present study showed that SOC and TN content influence the response of grain yield and NUE to the application of CRU. SOC is the largest carbon pool of terrestrial ecosystems and play an important role in ecosystem productivity [36]. Numerous studies consistently illustrated that fertilizer N application could improve SOC content and increase crop productivity [37,38]. The results of present study demonstrated that the positive effects of grain yield to CRU application tended to decrease with the increase in SOC content (Fig 5), suggesting that utilization of CRU into infertile soil generated greater yield increase. This might be due to the fact that CRU can greatly improve the fertility of infertile soil. Soil TN content is the main factor restricting crop production generally especially for the soil with low TN content. [39]. And grain yield might be inherently low in soil with extremely low TN content [40]. Consequently, grain yield and NUE response to CRU tended to be greater in the soil with extremely low TN content.

## Conclusion

Based on this meta-analysis, CRU application significantly increased SOC, TN, and AN. Grain yield and NUE showed positive response to the application of CRU which was attributed to the slow N release characteristics of CRU. The increase of NUE and grain yield by the application of CRU were greatest when the content of SOC and TN were extremely low, indicating that it was effective to improve grain production of infertile soil by applying CRU. We concluded that applying CRU should be promoted for improving grain production and NUE, especially for the soil with extremely low SOC and TN.

## Supporting information

S1 PRISMA checklist (DOCX)

S1 Table Basic information of the compiled 89 publications extracted for this meta-analysis. (DOCX)

S2 Table Quality assessment of included studies. (DOCX)
