# Supplementary material for: Application of controlled release urea improved grain yield and nitrogen use efficiency: A meta-analysis
Source: PLoS One. 2020 Oct 29;15(10):e0241481. doi: 10.1371/journal.pone.0241481 (PMC7595396; doi:10.1371/journal.pone.0241481)
Supplement: S1 Table — (DOCX) [file pone.0241481.s002.docx]

**Supporting information for**

**Application of controlled release urea crop improved grain yield and nitrogen use efficiency: A meta-analysis**

Shuhao Zhu#, Liyuan Liu#, Yan Xu, Yanying Yang, Rongguang Shi*

Agro-Environmental Protection Institute Ministry of Agriculture, Tianjin, 300110, China

# The first two authors contributed equally to this work

Table.S1

Basic information of the compiled 89 publications extracted for this meta-analysis.

| **NO** | **Location** | | **MAP （mm）** | **MAT (**°C**)** | **DOI** |
| --- | --- | --- | --- | --- | --- |
| 1 | 35°57′N | 117°01′E | 687.7 | 12.8 | DOI:10.16175/j.cnki.1009-4229.2016.10.020 |
| 2 | 36°10′N | 117°09′E | 512 | 13.2 | http://dx.doi.org/10.1016/j.eja.2016.01.010 |
| 3 | 35°12′N | 107°40′E | 581.2 | 9.25 | http://dx.doi.org/10.1016/j.still.2015.11.003 |
| 4 | 34°20′N | 108°24′E | 632 | 13 | doi: 10.1016/S2095-3119(16)61487-9 |
| 5 | 36°09′N | 117°09′E | 512 | 13.2 | http://dx.doi.org/10.1016/j.fcr.2016.08.004 |
| 6 | 36°57′N | 117°59′E | 587 | 12.5 | http://dx.doi.org/10.1016/j.fcr.2017.02.009 |
| 7 | 30°12′N | 119°53′E | 1490 | 17.2 | http://dx.doi.org/10.1016/j.fcr.2012.12.003 |
| 8 | 30°21′N | 112°09′E | 927 | 16.7 | http://dx.doi.org/10.1016/j.agee.2015.09.008 |
| 9 | 30°29′N | 111°45′E | 1041.8 | 16.5 | http://dx.doi.org/10.1016/j.fcr.2015.09.003 |
| 10 | 29°19′N | 119°43′E | 1489 | 18.2 | https://doi.org/10.1016/j.fcr.2018.11.013 |
| 11 | 29°10′N | 120°54′E | 1411.7 | 17 | doi: 10.1016/S2095-3119(18)62052-0 |
| 12 | 32°38′N | 110°37′E | 769.6 | 15.4 | http://dx.doi.org/10.1016/j.fcr.2014.08.014 |
| 13 | 31°53′N | 117°10′E | 1000 | 15.7 | http://dx.doi.org/10.1016/j.fcr.2017.07.005 |
| 14 | 29°01′N | 119°27′E | 1400 | 17.9 | https://doi.org/10.1016/j.fcr.2019.02.012 |
| 15 | 38°07′N | 106°17′E | 230.7 | 8.8 | doi: 10.1016/S2095-3119(14)60952-7 |
| 16 | 32°00′N | 119°32′E | 882 | 16.4 | https://doi.org/10.1016/j.agee.2018.06.023 |
| 17 | 30°21′N | 112°09′E | 1041.8 | 16.5 | https://doi.org/10.1016/j.geoderma.2017.11.033 |
| 18 | 35°08′N | 116°53′E | 727.1 | 13.7 | https://doi.org/10.1016/j.jenvman.2018.05.010 |
| 19 | 31°22′N | 119°47′E | 1294.5 | 16.1 | https://doi.org/10.1016/j.apsoil.2019.103469 |
| 20 | 34°46′N | 117°08′E | 700 | 14.2 | http://dx.doi.org/10.1016/j.agwat.2013.03.011 |
| 21 | 36°10′N | 117°04′E | 512 | 13.2 | https://doi.org/10.1016/j.agwat.2019.105834 |
| 22 | 29°01′N | 119°27′E | 1424 | 17.5 | http://dx.doi.org/10.1016/j.eja.2017.07.013 |
| 23 | 38°54′N | 100°21′E | 146 | 7.38 | https://doi.org/10.1016/j.fcr.2019.107624 |
| 24 | 36°09′N | 117°09′E | 683.2 | 13.2 | https://doi.org/10.1016/j.still.2019.104438 |
| 25 | 47°10′N | 127°10′E | 600 | 2.5 | DOI:10.16170/j.cnki.1673-6737.2009.05.018 |
| 26 | 36°57′N | 118°12′E | 639.7 | 13.2 | DOI: 10．14083 /j．issn．1001－4942．2020．02． 012 |
| 27 | 36°18′N | 117°29′E | 683.2 | 13.2 | DOI: 10．14083 /j． issn．1001－4942．202002． 013 |
| 28 | 36°47′N | 116°42′E | 547.5 | 12.9 | DOI: 1007-6220（2019）11-0049-03 |
|  | 35°38′N | 116°41′E | 733 | 13.6 |  |
| 29 | 41°52′N | 115°49′E | 375 | 1 | CNKI:SUN:CDXU.0.2012-05-023 |
| 30 | 30°42′N | 103°51′E | 896.1 | 15.8 | DOI: 10.3724/SP.J.1006.2014.00859 |
| 31 | 38°39′N | 106°08′E | 203 | 8.3 | CNKI:SUN:ZNTB.0.2015-17-031 |
| 32 | 28°25′N | 115°51′E | 1600 | 17.5 | DOI:10.13758/j.cnki.tr.2018.01.006 |
| 33 | 36°18′N | 119°58′E | 686.5 | 14 | CNKI:SUN:ZNTB.0.2013-27-021 |
| 34 | 40°12′N | 116°14′E | 550.3 | 11.8 | CNKI:SUN:ZWYF.0.2008-04-025 |
| 35 | 36°46′N | 117°26′E | 600.8 | 12.8 | DOI：10.13870/j.cnki.stbcxb.2020.02.033 |
| 36 | 25°04′N | 110°18′E | 1974 | 19.4 | DOI:10.16768/j.issn.1004-874x.2016.02.013 |
| 37 | 32°07′N | 119°50′E | 1000 | 15.1 | doi: 10. 11674 / zwyf． 14455 |
| 38 | 38°07′N | 106°17′E | 192.9 | 8.9 | doi: 10.13254/j.jare.2013.0157 |
| 39 | 38°07′N | 106°17′E | 192.9 | 8.9 | DOI：10.13254/j.jare.2013.0157 |
| 40 | 30°53′N | 121°23′E | 1162 | 15.7 | DOI：10.3969/j.issn.1006-8082.2015.04.021 |
| 41 | 29°54′N | 119°53′E | 1350 | 15.8 | DOI： CNKI:SUN:TRTB.0.2013-01-034 |
| 42 | 45°33′N | 127°01′E | 530 | 3.5 | DOI： CNKI:SUN:TRTB.0.2015-03-025 |
|  | 45°26′N | 126°22′E | 481 | 4.4 |  |
| 43 | 22°03′N | 112°56′E | 1936 | 21.8 | DOI：casb15080078 |
| 44 | 36°11′N | 117°07′E | 683.2 | 13.2 | DOI :10.13287/j .1001 -9332.2009.0403 |
| 45 | 32°56′N | 114°09′E | 1004.4 | 14.8 | DOI：1004-3918（2011）11-1331-04 |
|  | 33°03′N | 114°00′E | 1004.4 | 14.8 |  |
| 46 | 36°09′N | 117°09′E | 638 | 156 | DOI： 10.19336/j.cnki.trtb.2016.04.24 |
| 47 | 29°50′N | 121°40′E | 1538.8 | 16.2 | DOI：10.13870/j.cnki.stbcxb.2020.01.035 |
| 48 | 32°56′N | 114°09′E | 1004.4 | 14.8 | DOI :10.15933/j .cnki .1004 -3268.2009.08.026 |
|  | 33°03′N | 114°00′E | 1004.4 | 14.8 |  |
| 49 | 32°56′N | 114°09′E | 1004.4 | 14.8 | DOI： CNKI:SUN:ZNTB.0.2009-12-037 |
|  | 33°03′N | 114°00′E | 1004.4 | 14.8 |  |
| 50 | 35°10′N | 113°43′E | 573.5 | 14.5 | https://doi.org/10.13304/j.nykjdb.2019.0959 |
| 51 | 35°40′N | 114°17′E | 664.9 | 14.5 | DOI:10.16035/j.issn.1001-7283.2014.04.023 |
|  | 34°57′N | 112°59′E | 625 | 14.5 |  |
| 52 | 28°24′N | 115°52′E | 1662.5 | 17.8 | DOI:10.19386/j.cnki.jxnyxb.2016.05.007 |
|  | 26°25′N | 115°17′E | 1522.3 | 18.9 |  |
| 53 | 36°46′N | 117°26′E | 600.8 | 12.8 | doi: 10.11674/zwyf.19082 |
| 54 | 35°09′N | 113°48′E | 573.4 | 14 | doi: 10. 11674 / zwyf．14526 |
| 55 | 45°44′N | 126°43′E | 530 | 3.5 | DOI：10.3969/j.issn.1006-8082.2020.01.015 |
| 56 | 36°14′N | 115°54′E | 523.9 | 13.9 | DOI:10.13287/j.1001-9332.20140409.007 |
| 57 | 31°27′N | 120°25′E | 1100 | 15.7 | doi: 10．3969 /j．issn．1000-4440．2018．04．010 |
| 58 | 40°37′N | 111°36′E | 379.4 | 7.2 | DOI: 10．16853/j．cnki．1009-3575．2019． 01． 004 |
| 59 | 27°44′N | 108°12′E |  | 17.3 | DOI:10.13605/j.cnki.52-1065/s.2015.s1.002 |
| 60 | 28°25′N | 115°51′E | 1600 | 17.5 | DOI: 10.13758/j.cnki.tr.2018.01.006 |
| 61 | 43°49′N | 125°22′E | 593.8 | 4.9 | DOI: 10.13597/j.cnki.maize.science.20170419 |
| 62 | 30°42′N | 103°51′E | 896.1 | 15.8 | doi: 10. 11674 /zwyf．2013．0503 |
| 63 | 31°32′N | 120°06′E | 1112.3 | 15.6 | DOI： CNKI:SUN:TRXB.0.2011-05-012 |
| 64 | 36°13′N | 111°34′E | 500 | 12.9 | doi: 10．7668 / hbnxb．2017．01．026 |
| 65 | 36°51′N | 115°0′E | 542.8 | 13 | DOI: 10.19336/j.cnki.trtb.2018.01.20 |
| 66 | 32°39′N | 119°43′E | 978.7 | 14.9 | doi：10.11838/sfsc.1673-6257.18382 |
| 67 | 47°02′N | 125°19′E | 417.2 | 4 | DOI: 10．13287 /j．1001－9332．202001．021 |
| 68 | 32°36′N | 120°08′E | 991.7 | 14.5 | DOI: 10.3724/SP.J.1006.2017.00730 |
| 69 | 36°46′N | 117°26′E | 600.8 | 12.8 | doi: 10. 11838 /sfsc. 20180102 |
| 70 | 31°15′N | 120°57′E | 1097.1 | 15.5 | DOI： 10.3969/j.issn.1007-4929.2014.05.003 |
| 71 | 36°15′N | 117°15′E | 687.95 | 13.42 | DOI:10.13870/j.cnki.stbcxb.2014.02.030 |
| 72 | 38°04′N | 106°16′E | 174 | 8.5 | doi: 10. 11838 / sfsc. 20180510 |
| 73 | 45°49′N | 128°48′E | 579.7 | 2.4 | DOI： CNKI:SUN:GGPS.0.2018-10-010 |
| 74 | 29°59′N | 119°37′E | 1360.7 | 16.8 | DOI：0439－8114（2014）06－1289－05 |
| 75 | 36°41′N | 116°54′E | 685 | 14.2 | DOI:10.13870/j.cnki.stbcxb.2014.01.044 |
| 76 | 38°57′N | 115°56′E | 529.7 | 12.2 | DOI:10.16318/j.cnki.hbnykx.2012.02.022 |
|  | 38°51′N | 115°58′E | 529.7 | 12.2 |  |
| 77 | 37°27′N | 116°19′E | 547.5 | 12.9 | DOI: 10．13287 /j．1001－9332．201904．019 |
| 78 | 43°48′N | 125°24′E | 570.3 | 4.7 | doi:10.11654/jaes.2016-0412 |
| 79 | 30°05′N | 104°34′E | 965.8 | 16.8 | DOI:10.13870/j.cnki.stbcxb.2012.06.052 |
| 80 | 43°34′N | 125°53′E | 525 | 4.5 | doi:10.11654/jaes.2017-1762 |
| 81 | 35°02′N | 114°43′E | 656.3 | 14 | DOI: 10.13758/j.cnki.tr.2016.01.008 |
| 82 | 23°23′N | 104°13′E | 1187.8 | 18.4 | DOI：10.3969/j.issn.1006-8082.2015.04.019 |
|  | 25°01′N | 103°40′E | 950 | 14.7 |  |
| 83 | 36°11′N | 117°07′E | 803.7 | 13.2 | doi: 10.11674 / zwyf．2015.0231 |
| 84 | 31°52′N | 117°14′E | 1000 | 15.7 | doi: 10. 11674 /zwyf．2013.0405 |
| 85 | 36°11′N | 117°07′E | 803.7 | 13.2 | DOI:10.13287/j.1001-9332.2012.0009 |
| 86 | 36°09′N | 117°09′E | 700 | 12.8 | doi: 10.11674/zwyf.18285 |
| 87 | 28°23′N | 113°53′E | 1852 | 17.5 | doi: casb18080093 |
| 88 | 27°00′N | 106°07′E | 1087.5 | 14.2 | doi: 1001-3601( 2020) 02-0040-0015-06 |
| 89 | 35°40′N | 114°17′E | 664.9 | 14.5 | DOI:10.16445/j.cnki.1000-2340.2017.04.002 |
|  | 34°08′N | 113°08′E | 711.1 | 14.7 |  |
