# Supplementary material for: Application of controlled release urea improved grain yield and nitrogen use efficiency: A meta-analysis
Source: PLoS One. 2020 Oct 29;15(10):e0241481. doi: 10.1371/journal.pone.0241481 (PMC7595396; doi:10.1371/journal.pone.0241481)
Supplement: S2 Table — (DOCX) [file pone.0241481.s003.docx]

**Supporting information for**

**Application of controlled release urea crop improved grain yield and nitrogen use efficiency: A meta-analysis**

Shuhao Zhu#, Liyuan Liu#, Yan Xu, Yanying Yang, Rongguang Shi*

Agro-Environmental Protection Institute Ministry of Agriculture, Tianjin, 300110, China

# The first two authors contributed equally to this work

**Table. S2 Quality assessment of included studies**

| No. | Study | Define the source of information | Inclusion criteria | Blind method | Random allocation | Treatment representativeness | Data integrity | Study continuity | Selective reporting | Total |
| --- | --- | --- | --- | --- | --- | --- | --- | --- | --- | --- |
|  |  |  |  |  |  |  |  |  |  |  |
| 1 | Shi et al. 2016 | 1 | 1 | 0 | 1 | 1 | 1 | 0 | 1 | 6 |
| 2 | Guo et al. 2016 | 1 | 1 | 0 | 1 | 0 | 1 | 1 | 0 | 5 |
| 3 | Liu et al. 2016 | 1 | 1 | 0 | 1 | 0 | 0 | 1 | 0 | 4 |
| 4 | Yin et al. 2017 | 1 | 1 | 0 | 1 | 1 | 1 | 1 | 1 | 7 |
| 5 | Zheng et al. 2016 | 1 | 1 | 0 | 1 | 0 | 1 | 1 | 1 | 6 |
| 6 | Zheng et al. 2017 | 1 | 1 | 0 | 1 | 0 | 1 | 1 | 1 | 6 |
| 7 | Ye et al. 2013 | 1 | 1 | 0 | 1 | 1 | 1 | 1 | 1 | 7 |
| 8 | Wang et al. 2016 | 1 | 1 | 0 | 1 | 0 | 0 | 1 | 0 | 4 |
| 9 | Geng et al. 2015 | 1 | 1 | 0 | 1 | 1 | 1 | 1 | 1 | 7 |
| 10 | Sun et al. 2019 | 1 | 1 | 0 | 1 | 1 | 0 | 1 | 0 | 5 |
| 11 | Wei et al. 2018 | 1 | 1 | 0 | 1 | 0 | 0 | 1 | 0 | 4 |
| 12 | Tao et al. 2014 | 1 | 1 | 0 | 1 | 1 | 0 | 0 | 1 | 5 |
| 13 | Yang et al. 2017 | 1 | 1 | 0 | 1 | 0 | 0 | 1 | 0 | 4 |
| 14 | Mi et al. 2019 | 1 | 1 | 0 | 1 | 1 | 1 | 1 | 1 | 7 |
| 15 | Zhang et al. 2016 | 1 | 1 | 0 | 1 | 1 | 1 | 1 | 0 | 6 |
| 16 | Ke et al. 2018 | 1 | 1 | 0 | 1 | 1 | 1 | 1 | 1 | 7 |
| 17 | Li et al. 2018 | 1 | 1 | 0 | 1 | 0 | 0 | 1 | 0 | 4 |
| 18 | Zhang et al. 2018 | 1 | 1 | 0 | 1 | 1 | 1 | 1 | 1 | 7 |
| 19 | Lan et al. 2020 | 1 | 1 | 0 | 1 | 1 | 0 | 1 | 0 | 5 |
| 20 | Tian et al. 2013 | 1 | 1 | 0 | 1 | 1 | 0 | 0 | 0 | 4 |
| 21 | Li et al. 2020 | 1 | 1 | 0 | 1 | 1 | 0 | 1 | 0 | 5 |
| 22 | Mi et al. 2017 | 1 | 1 | 0 | 1 | 1 | 1 | 1 | 1 | 7 |
| 23 | Lyu et al. 2019 | 1 | 1 | 0 | 1 | 1 | 1 | 1 | 1 | 7 |
| 24 | Li et al. 2020 | 1 | 1 | 0 | 1 | 1 | 1 | 1 | 0 | 6 |
| 25 | Liu et al. 2009 | 1 | 1 | 0 | 1 | 1 | 1 | 1 | 1 | 7 |
| 26 | Jiang et al. 2020 | 1 | 1 | 0 | 1 | 1 | 1 | 0 | 1 | 6 |
| 27 | Liu et al. 2020 | 1 | 1 | 0 | 1 | 1 | 1 | 0 | 1 | 6 |
| 28 | Zhu et al. 2019 | 1 | 1 | 0 | 1 | 1 | 0 | 0 | 0 | 4 |
| 29 | Wang et al. 2012 | 1 | 1 | 0 | 1 | 1 | 1 | 0 | 0 | 5 |
| 30 | Peng et al. 2014 | 1 | 1 | 0 | 1 | 1 | 0 | 1 | 0 | 5 |
| 31 | Zhao et al. 2015 | 1 | 1 | 0 | 1 | 1 | 0 | 0 | 0 | 4 |
| 32 | Hou et al. 2016 | 1 | 1 | 0 | 1 | 1 | 1 | 1 | 1 | 7 |
| 33 | Jiang et al. 2013 | 1 | 1 | 0 | 1 | 0 | 1 | 0 | 0 | 4 |
| 34 | Xiao et al. 2008 | 1 | 1 | 0 | 1 | 1 | 0 | 1 | 0 | 5 |
| 35 | Zhang et al. 2020 | 1 | 1 | 0 | 1 | 1 | 1 | 1 | 1 | 7 |
| 36 | Shi et al. 2016 | 1 | 1 | 0 | 1 | 1 | 0 | 0 | 0 | 4 |
| 37 | Xu et al. 2016 | 1 | 1 | 0 | 1 | 0 | 1 | 1 | 1 | 6 |
| 38 | Liu et al. 2014 | 1 | 1 | 0 | 1 | 1 | 0 | 0 | 0 | 4 |
| 39 | Zhang et al. 2012 | 1 | 1 | 0 | 1 | 1 | 1 | 0 | 1 | 6 |
| 40 | Sun et al. 2015 | 1 | 1 | 0 | 1 | 0 | 1 | 1 | 1 | 6 |
| 41 | Yang et al. 2013 | 1 | 1 | 0 | 1 | 1 | 1 | 0 | 1 | 6 |
| 42 | Ji et al. 2015 | 1 | 1 | 0 | 1 | 1 | 1 | 0 | 1 | 6 |
| 43 | Zhang et al. 2016 | 1 | 1 | 0 | 1 | 0 | 0 | 0 | 1 | 4 |
| 44 | Zhao et al. 2009 | 1 | 1 | 0 | 1 | 1 | 1 | 1 | 1 | 7 |
| 45 | Sun et al. 2011 | 1 | 1 | 0 | 1 | 1 | 1 | 0 | 1 | 6 |
| 46 | Guo et al. 2016 | 1 | 1 | 0 | 1 | 1 | 1 | 0 | 1 | 6 |
| 47 | Jin et al. 2020 | 1 | 1 | 0 | 1 | 1 | 0 | 0 | 0 | 4 |
| 48 | Sun et al. 2009 | 1 | 1 | 0 | 1 | 1 | 1 | 0 | 1 | 6 |
| 49 | Sun et al. 2009 | 1 | 1 | 0 | 1 | 1 | 1 | 0 | 1 | 6 |
| 50 | Yin et al. 2020 | 1 | 1 | 0 | 1 | 1 | 1 | 0 | 1 | 6 |
| 51 | Liu et al. 2014 | 1 | 1 | 0 | 1 | 1 | 0 | 0 | 0 | 4 |
| 52 | Feng et al. 2016 | 1 | 1 | 0 | 1 | 1 | 1 | 0 | 1 | 6 |
| 53 | Zhang et al. 2020 | 1 | 1 | 0 | 1 | 1 | 1 | 0 | 1 | 6 |
| 54 | Zhou et al. 2016 | 1 | 1 | 0 | 1 | 1 | 1 | 1 | 1 | 7 |
| 55 | Lv et al. 2020 | 1 | 1 | 0 | 1 | 1 | 0 | 0 | 0 | 4 |
| 56 | Si et al. 2014 | 1 | 1 | 0 | 1 | 1 | 1 | 0 | 0 | 5 |
| 57 | Liu et al. 2018 | 1 | 1 | 0 | 1 | 1 | 0 | 0 | 0 | 4 |
| 58 | Wei et al. 2019 | 1 | 1 | 0 | 1 | 1 | 1 | 0 | 1 | 6 |
| 59 | Hou et al. 2015 | 1 | 1 | 0 | 1 | 1 | 0 | 0 | 0 | 4 |
| 60 | Hou et al. 2018 | 1 | 1 | 0 | 1 | 1 | 1 | 1 | 1 | 7 |
| 61 | Li et al. 2017 | 1 | 1 | 0 | 1 | 1 | 0 | 0 | 0 | 4 |
| 62 | Peng et al. 2013 | 1 | 1 | 0 | 1 | 1 | 0 | 0 | 0 | 4 |
| 63 | Xue et al. 2011 | 1 | 1 | 0 | 1 | 1 | 1 | 0 | 1 | 6 |
| 64 | Yang et al. 2017 | 1 | 1 | 0 | 1 | 1 | 1 | 1 | 1 | 7 |
| 65 | Zhang et al. 2018 | 1 | 1 | 0 | 1 | 1 | 0 | 1 | 0 | 5 |
| 66 | Deng et al. 2019 | 1 | 1 | 0 | 1 | 1 | 1 | 0 | 1 | 6 |
| 67 | Wu et al. 2020 | 1 | 1 | 0 | 1 | 0 | 0 | 1 | 0 | 4 |
| 68 | Wei et al. 2017 | 1 | 1 | 0 | 1 | 0 | 1 | 0 | 0 | 4 |
| 69 | Xu et al. 2018 | 1 | 1 | 0 | 1 | 1 | 0 | 0 | 0 | 4 |
| 70 | Liu et al. 2014 | 1 | 1 | 0 | 1 | 0 | 1 | 1 | 0 | 5 |
| 71 | Feng et al. 2014 | 1 | 1 | 0 | 1 | 1 | 0 | 0 | 0 | 4 |
| 72 | Hong et al. 2018 | 1 | 1 | 0 | 1 | 1 | 0 | 0 | 0 | 4 |
| 73 | Liu et al. 2018 | 1 | 1 | 0 | 1 | 1 | 1 | 0 | 1 | 6 |
| 74 | Wang et al. 2014 | 1 | 1 | 0 | 1 | 1 | 0 | 0 | 0 | 4 |
| 75 | Jiang et al. 2014 | 1 | 1 | 0 | 1 | 1 | 1 | 0 | 1 | 6 |
| 76 | Ru et al. 2012 | 1 | 1 | 0 | 1 | 1 | 1 | 1 | 1 | 7 |
| 77 | Zhang et al. 2019 | 1 | 1 | 0 | 1 | 0 | 0 | 1 | 0 | 4 |
| 78 | Yan et al. 2016 | 1 | 1 | 0 | 1 | 1 | 1 | 0 | 1 | 6 |
| 79 | Luo et al. 2012 | 1 | 1 | 0 | 1 | 1 | 0 | 0 | 0 | 4 |
| 80 | Song et al. 2018 | 1 | 1 | 0 | 1 | 1 | 0 | 0 | 0 | 4 |
| 81 | Liang et al. 2016 | 1 | 1 | 0 | 1 | 1 | 0 | 1 | 0 | 5 |
| 82 | Li et al. 2015 | 1 | 1 | 0 | 1 | 1 | 0 | 0 | 0 | 4 |
| 83 | Liu et al. 2015 | 1 | 1 | 0 | 1 | 1 | 1 | 0 | 1 | 6 |
| 84 | Li et al. 2013 | 1 | 1 | 0 | 1 | 1 | 0 | 0 | 0 | 4 |
| 85 | Ma et al. 2012 | 1 | 1 | 0 | 1 | 1 | 1 | 0 | 1 | 6 |
| 86 | Dong et al. 2019 | 1 | 1 | 0 | 1 | 0 | 1 | 1 | 0 | 5 |
| 87 | Hu et al. 2019 | 1 | 1 | 0 | 1 | 0 | 1 | 0 | 1 | 5 |
| 88 | Xie et al. 2020 | 1 | 1 | 0 | 1 | 1 | 1 | 0 | 1 | 6 |
| 89 | Zhang et al. 2017 | 1 | 1 | 0 | 1 | 1 | 0 | 0 | 0 | 4 |
